# An Examination of Growing Trends in Land Tenure and Conservation Practice Adoption: Results from a Farmer Survey in Iowa

**DOI:** 10.1007/s00267-015-0619-5

**Published:** 2015-10-30

**Authors:** Sarah Varble, Silvia Secchi, Caroline Gottschalk Druschke

**Affiliations:** Southern Illinois University, Faner Hall, Mail Code 4541, Carbondale, IL 62901 USA; University of Rhode Island, 105 Coastal Institute, 1 Greenhouse Road, Kingston, RI 02881 USA

**Keywords:** Farmland tenure, Communication, Conservation practice adoption, Conservation outreach

## Abstract

Tenants and part-owners are farming an increasing number of acres in the United States, while full-owners are farming fewer acres. This shift in ownership is a potential cause for concern because some previous research indicated that tenant and part-owner farmers were less likely to adopt conservation practices than farmers who owned the land they farmed. If that trend persists, ownership changes would signal a national drop in conservation adoption. Here we examine this issue using a survey of agricultural operators in the Clear Creek watershed in Iowa, a state with intensive agricultural production. We compare adoption of conservation practices, and preferences for conservation information sources and communication channels, between farmers who rent some portion of the land they farm (tenants and part-owners) and farmers who own all of the land they farm (full-owners). We find that renters are more likely to practice conservation tillage than full-owners, though they are less likely to rotate crops. In addition, renters report using federal government employees (specifically, Natural Resource Conservation Service and Farm Service Agency) as their primary sources of conservation information, while full-owners most frequently rely on neighbors, friends, and County Extension. These findings are significant for conservation policy because, unlike some past research, they indicate that renters are not resistant to all types of conservation practices, echoing recent studies finding an increase in conservation adoption among non-full-owners. Our results emphasize the importance of government conservation communication and can inform outreach efforts by helping tailor effective, targeted conservation strategies for owners and renters.

## Introduction

Agricultural land ownership in the U.S. is changing. In past years, full-owners (agricultural operators who own 100 % of the land they farm) farmed a majority of American farmland. But due to shifts in ownership and changes in farm size over the past three decades, renters (including tenant farmers who rent 100 % of the land they farm, and part-owners who both own and rent farmland) now farm an increasing number of acres, especially in the agriculturally productive Midwestern United States. In 2012, more than 354 million acres across the United States were rented to agricultural operators for animal or plant production (National Agricultural Statistics Service 2014).

**Fig. 1:**
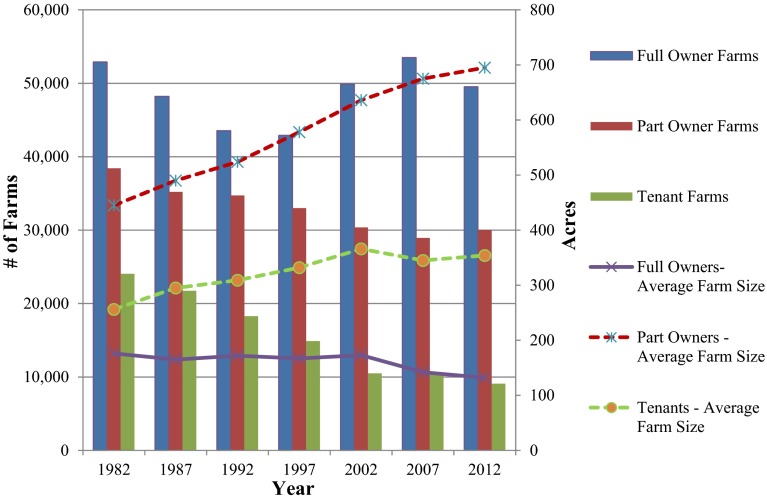
Iowa 1982–2012: number of farms and average farm size

Nationally, rented farmland has decreased from 40 to 38 %; however, in the Midwestern United States, the numbers are increasing, and now a larger portion of farmland is managed by renters rather than owners. In Iowa, the leading state for corn production, 53 % of farmland (16 million acres) was farmed by renters in 2012, up from 48 % in 1982 (Bureau of the Census 1983; National Agricultural Statistics Service 2014). Meanwhile, the average farm size for part-owners and tenants has nearly doubled. The 2007 Census found that there are nearly 1500 part-owner and tenant operators who each farm more than 2000 acres in Iowa: a steep increase from the 238 part-owners and tenants who farmed over 2000 acres in 1982. Conversely, full-owners are farming fewer acres (Fig. 1). This farm size increase for part-owners and tenants is also a national trend, with part-owners and tenants operating 78 % of farms over 2000 acres nationally (National Agricultural Statistics Service 2014). These numbers illustrate a growing trend in Iowa and throughout the US: farm sizes are increasing for part-owners and tenants, while full-owners are, on average, farming fewer acres.

**Fig. 2:**
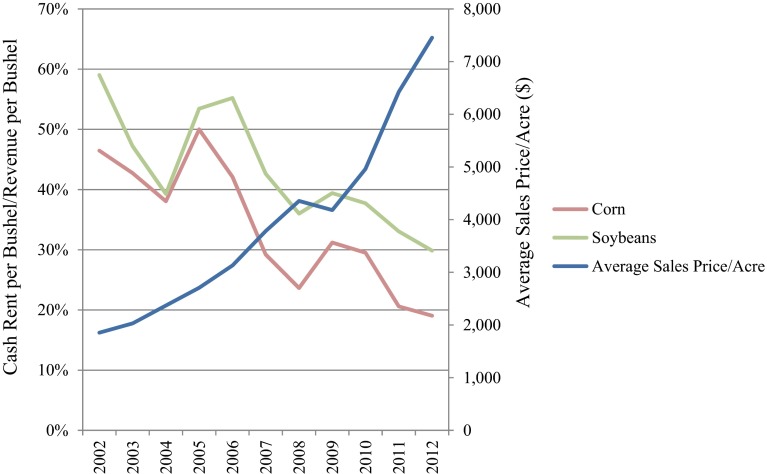
%Cash rent per revenue per bushel and average land sales prices (Iowa County, IA)

There is much speculation about why this trend is occurring. U.S. farmers need to increase production via an expansion in acreage to cover higher costs for equipment and other lumpy inputs like machinery, which cannot be acquired gradually and reach their minimum per unit cost at relatively large scales of production (Eastwood et al. 2010). High commodity prices may also be driving renters to farm more land to maximize profits, while higher land sales prices may make it difficult or even unfavorable for new farmers to purchase land, leaving them only to rent. In addition, a dip in cash rents for corn and soybeans in one of our surveyed counties makes renting potentially more lucrative than ownership alone. In Iowa County, included in our survey area, the percentage of revenue per bushel of corn paid in cash rent decreased from 46 % in 2002 to 19 % in 2012 (Fig. 2). Soybean revenue applied to rental prices decreased from 59 % of in 2002 to only 30 % in 2012 (Edwards 2012). Meanwhile, land sales prices quadrupled during the same time period (Duffy 2013). For farmers who are financially conservative or wary of a land price bubble, renting versus owning likely makes more financial sense. Renters who pay cash rents to owners may have lower net profits than owners; thus, they must farm more rented acres to see the same profits experienced by full-owner-operators. Financially savvy renters may also want to take advantage of economies of scale that can be gained by farming more acres.

The increase in both rentership and the acreage farmed by renters is significant for several reasons. First, some studies have shown that a divide exists between renters and owners in conservation practice adoption in the US and internationally. In the US, a limited number of studies have examined the farming and conservation practices of renters. There is little consensus on the impact of ownership, though all studies show conservation practice differences between the two groups, and those differences may be shifting over time. An early study found a negative correlation between full ownership and the adoption of minimum-till practices (Lee and Stewart 1983), but much of the subsequent research has found tenancy as negatively related to conservation adoption. Renters (both cash renters and share renters) have been found to be less likely to adopt long-term conservation practices, such as terraces or grassed waterways (Soule et al. 2000), and less likely to practice conservation tillage or crop rotation (Soule et al. 2000; Fraser 2004). But recent studies have indicated that this trend may be changing, finding that leasing was not a deterrent to the adoption of buffer strips (Tosakana et al. 2010) and showing that the percentage of renters versus owners was positively correlated to state-level application rates for conservation programs such as the Environmental Quality Incentives Program (Reimer et al. 2013).

Our research examines this trend, exploring whether renters are currently adopting certain types of conservation practices in higher numbers. Understanding the link between land tenancy and land management choices in the Upper Mississippi River Basin, as well as farmers’ preferred information sources and communication channels, is crucial both environmentally and economically because Corn Belt states like Iowa and Illinois are major producers of global agricultural commodities and contribute much of the non-point source pollution that causes Mississippi River and Gulf of Mexico hypoxia (Rabalais et al. 2002). Increased attention to the differences between agricultural owners and renters in the adoption of conservaton is more than warranted by the strong role of Midwestern agriculture in larger social-ecological systems, the decrease in conservation funding by the United States federal government in the last two farm bills due to budget constraints (USDA Economic Research Service 2014), the increase in agricultural intensity (Tilman et al. 2002), the increase in climate change related weather events (IPCC 2013; Rosenzweig et al. 2001), and the international impact of elevated nutrient loading in waterways (Rabalais et al. 2002).

The goal of this study is to explore the effect of tenure on conservation adoption, in light of recent rises in rentership in the Midwest and in farm size in the Midwest and nationally. To do that, we begin by introducing the study watershed and our survey instrument, and then review the connections between land tenure and crop rotation, tillage, information sources, and communication channels. We close with a discussion of the implications of our findings, and directions for future research.

## Materials and Methods

**Fig. 3:**
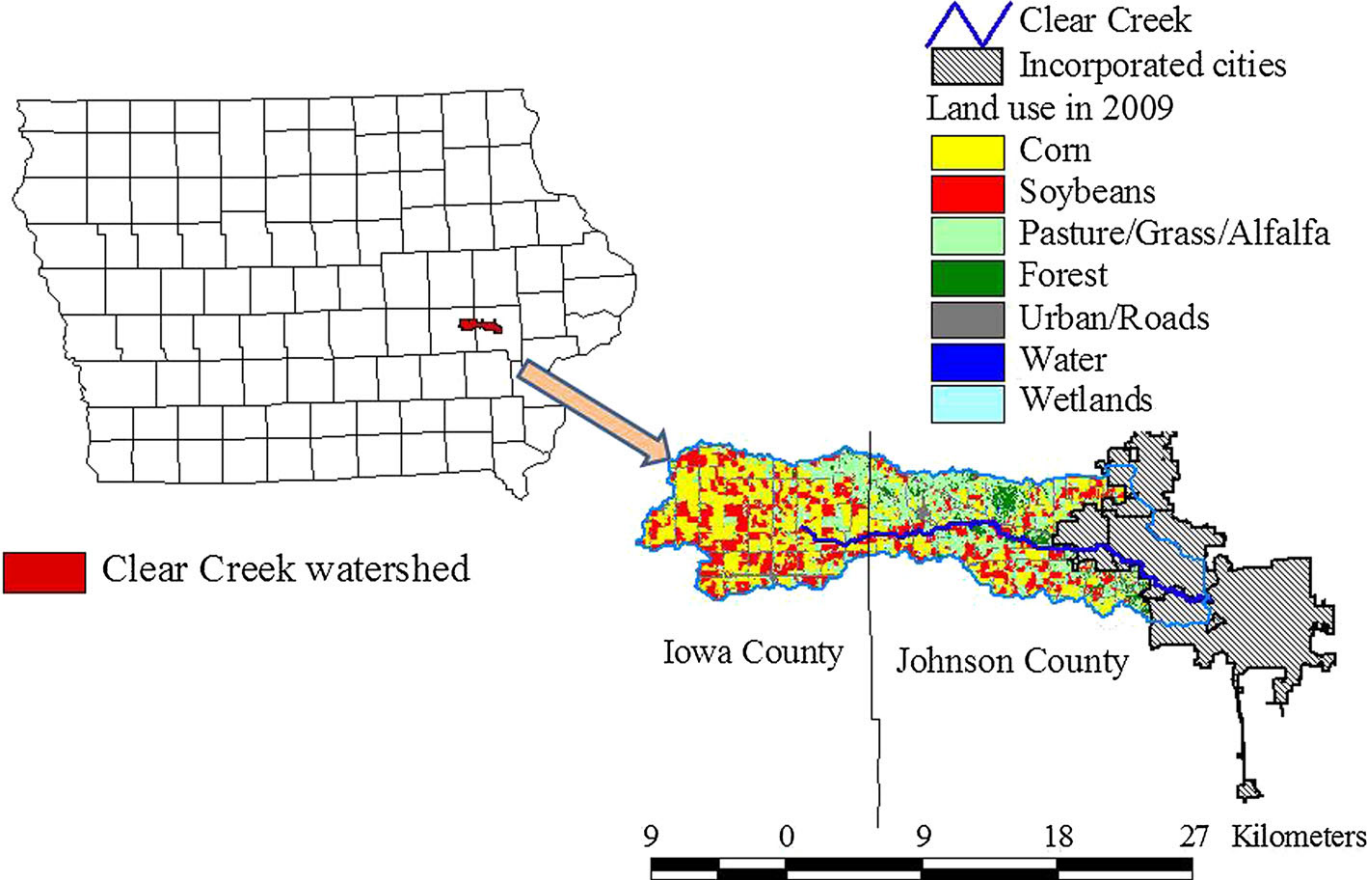
Clear Creek location and land use

The survey was administered in Iowa’s Clear Creek watershed in the US Corn Belt in 2010. In 2009, 29 % of the watershed’s 65,000 acres was in corn production, 22 % in soybean production, and 27 % in pasture, grass land, or alfalfa. In addition to agriculture, this watershed is 14 % urban, and includes the cities of Coralville, Iowa City, and North Liberty. Figure 3 shows the location of the watershed and its land use. Partly due to the non-point source pollution generated by agriculture, Clear Creek was listed on the United States Environmental Protection Agency’s (USEPA) Impaired Waters 303(d) list in 2004. According to a recent survey conducted by the USEPA, nutrient impairment is becoming a common trend as over half (55 %) of US waterways are impaired due to a variety of sources and do not support healthy populations of aquatic life (EPA 2013).

Watershed management groups are gaining popularity as a community-based approach to reducing water pollution. The Clear Creek watershed has a long-standing watershed management group that coordinates outreach with farmers in the area to reduce soil erosion and fertilizer runoff. As agricultural non-point source pollution becomes more of a problem globally, this type of approach has the potential to become a more common strategy for reducing pollution.

In addition to its water quality issues, overlaying georeferenced cropland and soils data, we found that more than half (57 %) of the land in this watershed is considered highly erodible, and 40 % is both highly erodible and highly suited for corn production (Iowa Cooperative Soil Survey 2003; USDA NASS Research and Development Division 2012). Current federal government policies mandate that farmers who farm highly erodible land must comply with standards that minimize erosion risk in order to qualify for subsidized crop insurance and, in previous Farm Bills, commodity subsidies. These policies are likely to impact farmers’ adoption of conservation practices in the watershed.

### Survey Description

The Institutional Review Board-approved survey was distributed by mail in April 2010 to all 998 rural landowners and farmers in the Clear Creek watershed. Names and addresses of landowners and farmers were obtained by cross referencing geographic information system county parcel data against a list of names and addresses supplied by the Farm Service Agency office in each county. Each landowner and farmer identified was mailed a 16-page survey and two reminder mailings. In cases where a husband and wife or relatives co-owned property, each person was mailed an individual survey. Thirty-one of the surveys were returned as undeliverable, and 397 partially or fully completely surveys were returned for a 41 % response rate (Druschke and Secchi 2014).

Of the full set of 397 respondents, 58 % were male, and the average age was 62, compared with 92 % male farmers statewide, with an average age of 57 (National Agricultural Statistics Service 2014). Since the focus of the study was the adoption of conservation practices by active farmers, only active farmers, both full-owners (farmers who own 100 % of the land they farm), and renters (both tenant and part-owner farmers who own 0–99 % of the land they farm) were included in the data analysis for this study. The result was a sample size of 143 (Table 2). Respondents farmed an average of 170 acres (with a standard deviation of 399 acres, and a range from 1 to 3040 acres) as compared to the 2012 average Iowa farm size of 345 acres (up from 331 acres in 2007) (National Agricultural Statistics Service 2009, 2014).

Survey respondents were broadly representative of other farmers in USDA’s Corn Belt states (Illinois, Indiana, Iowa, Missouri, and Ohio), making our particular findings relevant beyond the boundaries of the surveyed watershed (Table 1). Clear Creek’s full-owner survey respondents were older as in the rest of the region, and tenants were the youngest, though Clear Creek’s tenants were older than tenants in the rest of the region. Women were a minority in farming regardless of tenancy in Clear Creek and in the Corn Belt, but our respondents included a substantially higher percentage of women, particularly for part-owners and tenants. As in the overall region, part-owners in Clear Creek operated the largest farms. Tenants in Clear Creek who responded to the survey tended to have smaller farms than tenants in the Corn Belt. Finally, the crops grown as a percentage of total land are broadly representative of the region. There is more pasture and hay in Clear Creek than in Iowa and Illinois, for example, but about as much as the Missouri percentage.

**Table 1:** Clear Creek and the Corn Belt

Tenure and farmers characteristics	Full-owner	Part-owner and tenant
Age		
Clear Creek	63	57
Illinois	60	54
Indiana	57	53
Iowa	60	53
Missouri	60	54
Ohio	58	54
Gender: %women		
Clear Creek	30	34
Illinois	13	3
Indiana	13	4
Iowa	12	3
Missouri	14	5
Ohio	15	4
Total acres farmed		
Clear Creek	131	579
Illinois	111	711
Indiana	79	584
Iowa	132	616
Missouri	170	568
Ohio	80	405

Crops grown as percentage of total land	Corn %	Soybeans %	Hay, grassland pasture and range %
Clear Creek	29	22	27
Illinois	34	26	7
Indiana	24	24	10
Iowa	38	27	10
Missouri	7	12	28
Ohio	13	17	11

Sources: (U.S. Department of Agriculture National Agricultural Statistical Service 2012; Nickerson et al. 2011; USDA NASS 2010). The data on crops grown as percentage of total land area are from 2009, except the information on grassland, pasture and range which is from 2007, since USDA does not collect data on it annually

### Independent Variables

The sample was divided into two groups: full-owners and renters. Our survey had a low percentage of tenant-only farmers (2.3 %), which is consistent with the statistics from the USDA Census of Agriculture. Census data for Iowa and Johnson counties, the two counties that contain the Clear Creek watershed, show that 8.5 and 7.65 % of the counties’ farmers are tenants only, respectively. This percentage is even lower than the percent of tenant farmers in Iowa as a whole (11.2 %). The long-term downward trend of the number of tenant farmers compared to full-owners and part-owners (Fig. 1) suggests that this category may be obsolete in the next 10–20 years. Given this downward trend, the low number of tenants who completed our survey, and the approach taken in related literature, we decided to combine the part-owner and tenant categories into one encompassing “renter” category. There were 53 respondents categorized as “renters” and 86 categorized as “owners” (who we have been referring to up this point as full-owners). According to the 2007 US Census of Agriculture, 42.4 % of farmers in Iowa fall into our “renter” category and 57.6 % are categorized as owners (National Agricultural Statistics Service 2009). Our respondent group (excluding non-operators) was comparable to these numbers, with 38.2 % renters and 61.9 % owners.

Like the USDA Census, renters on average were younger than owners. They also had slightly less education. On par with the changing trends outlined above, the renters who completed the survey farmed more land than owners, had higher gross agricultural income, and had a higher percentage of household income from farming than owners (Tables 1, 2).

**Table 2:** Descriptive statistics about land ownership and rentership

Descriptive variable	Variable coding	Full-owners *N* = 86	Part-owners and tenants *N* = 53
Education	1 = some high school or less, 2 = high school diploma, 3 = vocational or tech diploma, 4 = some college but no degree, 5 = bachelor degree, 6 = graduate degree	3.63	3.41
Gross agricultural income	1 = $1–24,999 2 = $25K–99,999 3 = $100K–249,999 4 = $250K–499,999	1.83	3.02
%Household income from farming	1 = 1–25 % 2 = 26–50 % 3 = 51–75 % 4 = 76–100 %	1.74	2.42
Tillable acres owned		235.24	201.19
Own acres farmed		131.20	211.44
Leased acres farmed		0.00	367.81
Total acres farmed		131.20	579.25
Highly erodible acres farmed		79.00	371.09
Total # of information sources		2.95	3.14
Total communication channels		2.69	3.49

### Dependent Variables

#### Crop Rotation

The importance of understanding why and how renters and owners make decisions about conservation is heightened due to the increased demand for agricultural commodities, which is driven by world commodity markets and federal policies promoting corn ethanol. The U.S. consumption of biofuel, of which corn ethanol is a large component, has increased 11,000 million gallons since 2000 (U.S. Energy Information Administration 2012). As a result, the price per bushel of corn has more than doubled since the early 2000s (National Agricultural Statistics Service 2013). The higher prices have intensified corn production in the Midwest and many farmers have switched from a corn–soybean rotation to a continuous corn system to maximize profits, as our data illustrates for the renter category. This has negatively affected soil and environmental quality (Secchi et al. 2011). It is important to note that the bacteria on the roots of legumes (i.e., soybeans) fix nitrogen in the soil, and when the roots start to decay in the soil, nitrogen is released, thus providing nitrogen to the next year’s crop. This reduces the amount of nitrogen that must be applied by the farmer, and thus reduces input costs. Farmers who intensively plant corn can be considered short-term profit maximizers, and if it is true that renters are farming more acres in order to maximize profits, they would seem to fall into this category. It has long been argued that renters have a short-term connection with the land, especially if they lease year-to-year, and when making decisions about crop rotations, cash renters may put less weight on long-term net returns (Soule et al. 2000).

#### Tillage

Conservation tillage practices such as no-till, ridge-till, and mulch-till can increase the organic matter and water-holding capacity of the soil. They can also decrease soil erosion, fertilizer runoff, and required inputs like fuel and labor. Thus, they can be very beneficial to both the ecosystem and individual farmers. However, conservation tillage practices require different equipment, additional herbicide application, and, depending on the type of soil and climate, can actually reduce yields (Blanco-Canqui and Lal 2008). Additionally, it may take several years after adoption before the physical condition of the soil has improved enough to regain pre-adoption yields. Given the time lag before yields increase, several studies done in the 1980s found that owners were more likely to utilize conservation tillage than renters because renters had a short-term connection to the land and would not directly reap those longer-term benefits (Lynne et al. 1988; Belknap and Saupe 1988). Cash renters may worry less about future net returns (Soule et al. 2000). Thus, the logic goes, renters are focused on present profit in making decisions about conservation adoption.

However, there is some evidence to show that owner/operators are actually less likely to use conservation tillage than renters (Caswell et al. 2001) and may have lower rates of minimum-till adoption than other groups (Lee and Stewart 1983; Caswell et al. 2001). Renters may be equally or more concerned than owner/operators about soil health (Caswell et al. 2001), while owners may be less likely to utilize conservation tillage because of its aesthetic messiness (Ryan et al. 2003). There is a substantial amount of disagreement about the relationship between tenure and tillage.

#### Information Sources

A wealth of studies has shown the importance of knowledge dissemination for the adoption of a practice or technology. Knowledge sharing is an important aspect of adoption in the Diffusion of Innovation model (Rogers 2003), and access to information was a common denominator to the adoption of best management practices among 55 studies (Prokopy et al. 2008). Access to technical assistance and information sources were found to be significant predictors of adoption of conservation practices in three large Midwestern watersheds (Napier and Tucker 2001), where the Farm Service Agency, agri-chemical dealers, and the NRCS were the top three sources of information (Tucker and Napier 2002). The type of information source used by farmers determines what type of information they receive about conservation practices. If farmers receive information about a specific conservation practice, they will be more likely adopt that practice (Tucker and Napier 2002). Thus, if owners and renters use different sources for information, it might help to explain adoption differences.

#### Communication Channels

Besides information sources, communication channels are another important way of dispersing conservation information to farmers. They refer to the methods or media used to share information (Tucker and Napier 2002). The types of channels that farmers rely on impact their knowledge base and guide their farming decisions. In a 2002 multi-watershed study, a majority of farmers relied on farm magazines for information regarding conservation and farming practices (Tucker and Napier 2002). But technologies have changed dramatically since 2002 with internet use becoming much more prevalent. However, in rural areas, obtaining broadband, high-speed internet access can still be difficult. Thus, those farmers who rely on the internet for conservation information may be more advanced in their use of technologies and might be considered adoption leaders or innovators. The type of communication channel and number of channels used for information can provide some insight into how channels can be influential in conservation practice adoption. In addition, if a certain group (i.e., renters or owners) uses a channel more frequently than the other group, conservation agencies can make use of that channel to deliver tailored information that has a better chance of reaching the target audience, and, further, can consider how to interact with that particular group to make conservation information even more contextual and consequential.

### Crop Rotation and Land Tenure

While anecdotal evidence suggests that the relationship between rentership and connection to the land is somewhat complicated in the Clear Creek watershed because many renters are farming rented land that sits in close proximity to their homes and farms, we still hypothesized that renters would plant corn more intensively than owners.

#### **H1**

Renters will be more likely to plant corn more intensively than full-owners.

To determine whether owners or renters were more likely to participate in intensive corn production, we asked “What is your typical crop rotation on the land that you own/operate?” and “What is your typical crop rotation on the land you rent from others?” Answer choices included “Not applicable, corn/bean (all conventional till), hay, corn/corn/bean (all conventional till), corn/corn/bean (minimum-till corn, no-till bean), corn/bean (all no-till), CRP, and Other (specify).” Corn/bean responses were coded as “1”, and corn/corn/bean (intensive corn production) responses were coded as “2”. All other answers were coded “0”.

Due to the design of the questions, the responses were categorized into three groups based on the farmer categorization (renter or owner) and the land categorization (rented or owned). From our definition, “owners” only farm land that they own. However, renters (part-owners) farm land they rent *and* land they own. Thus, the categories analyzed are the following: owners; renters on the land they own and farm; and renters on the land they lease to farm. The results analyzed the farming practices of renters on land that they rent and on land that they own.

### Tillage and Land Tenure

Given the recent trends of continuous corn production, and the incompatibility of continuous corn production with no-till and minimum-till systems (Katsvairo and Cox 2000), it would seem that the profit-maximizing farmers who plant continuous corn would be less likely to utilize conservation tillage. Since we hypothesized that renters were more likely to plant continuous corn, we also hypothesized that renters would be less likely to utilize conservation tillage:

#### **H2**

Renters will be less likely to utilize conservation tillage than full-owners.

The same questions used to determine crop rotation were also used for tillage, and respondents were categorized into the same group types for this measurement. Both conservation tillage answer choices (no-till and minimum-till) were coded “1.” Conventional tillage answers were coded “2.” Table 3 shows means for each group.

**Table 3:** Tillage & rotation percentages for owners & renters

Practice	Renters (*n* = 53) (%)	Owners (*n* = 86) (%)
Planting rotation regime		
Corn/soybean	36	59
Corn/corn/soybean	64	41
Tillage regime		
Conservation tillage	79	74
Conventional tillage	21	26

### Information Sources and Land Tenure

In light of a lack of existing literature on differences in preferred information sources between owners and renters, we hypothesized that owners and renters would prefer the same sources of conservation information, and that those sources would mirror those highlighted in existing research (Farm Service Agency, agri-chemical dealers, and the NRCS) (Tucker and Napier 2002). Therefore:

#### **H3**

Owners and renters will both utilize representatives from the Farm Service Agency, agri-chemical dealers, and the Natural Resource Conservation Service as their main sources of conservation information.

Respondents were asked “Who is/are your main source(s) of information on conservation issues? (Check all that apply.)” Choices included: Natural Resources Conservation Service; Farm Service Agency; County Extension Service; Iowa State University Specialists; Local seed/chemical/fertilizer dealers; Neighbors and friends; Soil Conservation District Commissioners; Vocational Agriculture Instructors; Machinery dealers; Private consultants; Non-profit organizations; and Other, e.g., ASCS (please specify). If the respondent used a certain source, it was coded “1”; otherwise, it was coded “0.” Local seed/chemical/fertilizer dealers and machinery dealers were combined into one category: “agri-chem dealers.” Correlations were performed between the information sources and “owners” and “renters” to see if either group correlated highly with a specific information source.

### Communication Channels and Land Tenure

Due to a lack of existing literature about differences in communication channels between owners and renters, we hypothesized that printed materials would be an important source of conservation information for both owners and renters (Tucker and Napier 2002). Based on our previous observations and experience working with farmers in the watershed (Druschke 2013; Druschke and Secchi 2014), we suspected that renters would be more likely than owners to be early adopters or innovators, so we hypothesized that renters would rely on a wider group of sources than owners and would be more likely to rely on the internet:

#### **H4a**

Both renters and owners will rely on printed materials most often for information about conservation.

#### **H4b**

Renters will utilize more communication channels and will rely more on the internet than owners.

To determine which communication channels were utilized, respondents were asked, “How do you prefer to receive information about conservation issues? (Check all that apply.)” The choices included field demonstrations; county and local meetings; magazines; printed materials (brochures); trade shows and fairs; visual materials (slides, photographs); internet/webcasts/podcasts; television programs (DVDs, tapes); radio; and on-farm consultation. If a respondent utilized a specific channel, it was coded “1”; otherwise, it was coded “0.” A correlation between owners, renters, and communication channels was conducted to determine whether there were any significant relationships between communication channels and type of tenure.

## Results

### Crop Rotation and Land Tenure

A bivariate correlation showed a significant negative correlation between the independent variable “Owner/Renter” and crop rotations on the question that addressed crop rotations on land owned and farmed by the operator. A *t* test performed between the owner/renter independent variable and rotations also showed a significant difference (*t* (94) = 2.33, *P* = 0.022) between “Owners” and “Renters” on “land farmed by owner.” Based on these results, renters significantly plant corn more intensively on the land they own and farm than full-owners do. Renters also plant corn more intensively on rented land than owners; however, the difference is not significant. This shows partial support for hypothesis one: Renters will be more likely than full-owners to plant continuous corn (Table 3).

### Tillage and Land Tenure

Unlike the crop rotation results, full-owners are significantly more likely to practice conventional tillage than renters. *T* tests results on “land farmed by owner” show a significant difference between owners and renters (*t* (87.14) = −2.927, *P* = 0.004). However, there was little difference between the tillage practices of renters on the land they own and farm, and land they rent to farm. This does not support hypothesis two (renters will be less likely to utilize conservation tillage than full-owners), as renters are more likely to utilize conservation tillage than owners.

### Information Sources and Land Tenure

In partial support of H3, a correlation between owners/renters and information sources showed a significant relationship between NRCS and the owners/renters variable. An examination of the means (Table 4) shows that renters use the NRCS as an information source significantly more than owners do. Additionally, in further support of H3, both renters and owners also use the Farm Service Agency for information. Neither group relies heavily on agri-chem dealers for information.

**Table 4:** Correlations between information sources & channels used for conservation information and owners/renters

Information source	Owners (*n* = 86)	Renters (*n* = 53)	Correlation
NRCS	.395	.585	−0.185*
Farm Service Agency	.512	.585	−0.071
County Extension Service	.523	.528	−0.005
Iowa State University	.291	.283	0.008
Agri-chem dealer	.291	.359	−0.071
Neighbors & friends	.523	.377	0.142
Soil Conservation District Commissioner	.198	.226	−0.034
Ag instructors	.023	.038	−0.042
Non-profits	.070	.038	0.067
Field demonstrations (tours)	.22	.47	−0.26**
County and local meetings	.28	.43	−0.16
Magazines	.58	.60	−0.02
Printed materials (brochures)	.64	.60	0.04
Trade shows & fairs	.10	.25	−0.19*
Visual materials (slides, photos)	.06	.25	−0.27**
Internet, webcasts, podcasts	.17	.28	−0.13
TV programs (DVDs, tapes)	.22	.21	0.02
Radio	.17	.26	−0.11
On-farm consultation	.23	.23	0.01

* Correlation is significant at the 0.05 level (2-tailed)

** Correlation is significant at the 0.01 level (2-tailed)

The top three sources of information used by owners were neighbors and friends; the County Extension Service; and the Farm Service Agency. Renters used the NRCS, Farm Service Agency, and County Extension Service the most.

### Communication Channels and Land Tenure

Printed materials are the primary way that both renters and owners gain information, supporting H4a. The communication channels used most frequently by renters are (in order of popularity) printed materials; magazines; field demos; and meetings. Owners also rely heavily on printed materials, magazines, and meetings (in order of preference); however, on-farm consultations are also commonly used (Table 4).

On average, renters use significantly more channels of communication than owners, partially supporting H4b. A *t* test performed between the two groups showed that there was a significant difference (*t* (89.707) = 2.704, *P* = .008) between the groups. A correlation between the owner/renter variable and the communication channels showed significant negative relationships between owner/renter and field demos, trade shows, and visual material, meaning renters use these channels more frequently than owners.

## Discussion and Conclusions

Our results showed several significant differences between owners and renters. We hypothesized that renters would be more likely to plant an intensive corn rotation. This turned out to be true; however, renters also practice conservation tillage at a higher rate than owners. While renters plant corn more intensively, they also undertake the extra measures necessary to mitigate the impact of this practice by utilizing minimum tillage or no-till systems in a continuous corn regime. There may be a blend of economic and environmental factors at play. Renters must produce top-dollar crops to compensate for cash rents; meanwhile, they may be aware of the environmental and soil conservation benefits of minimal tillage regimes. Business savvy farmers balance both economic and environmental factors for maximum benefit. Adoption of conservation tillage practices is also driven by prices; if energy prices are high, adoption is in farmers’ best interests because they use less energy tilling fields. Agricultural producers must weigh the tradeoffs of boosting production and profits while maintaining environmental benefits, such as increasing soil organic matter and decreasing erosion. This balancing act can be difficult to achieve for many farmers, particularly since the policy and market landscapes keep changing at a rapid pace. Finally, renters can take advantage of decreased labor time associated with no-till practices.

The differences in tillage practices could also partially be attributed to the amount of highly erodible land (HEL) cultivated by each group. In our sample, renters on average cultivated 397 acres of HEL compared to an average of 79 HEL acres farmed by owners. In order to receive government subsidies, such as direct payments and crop disaster payments, farmers who planted crops on HEL had to be in conservation compliance to reduce erosion and runoff. Since the renters in our sample farmed more HEL than owners, utilization of conservation tillage might be an aspect of their compliance with the government regulations. Thus, our study shows that the coupling of conservation compliance with subsidized crop insurance is an effective way to increase conservation practices, especially since farmers want to ensure stable revenues and maximize profits. This is an important consideration for future policy creation, especially for policymakers crafting future farm bills.

The amount of HEL farmed by renters could also explain why renters use NRCS employees as a source of information more often than owners. The NRCS works with farmers who cultivate HEL land to help with compliance. A bivariate correlation shows a significant relationship (at the 0.01 level) between the amount of HEL farmed and the use of an NRCS employee as an information source. This means that farmers who farm more highly erodible land rely on NRCS agents more frequently. The finding that renters use the NRCS more often for conservation information is helpful in terms of information dissemination. Distribution of information by trusted individuals is a key part of innovation adoption, and since so many renters are utilizing NRCS employees for information, the NRCS can increase conservation practice adoption by continuing to cultivate relationships with landowners and renters and maximizing farmer-to-farmer networks for improving education about conservation practices. Many of the owners reported using friends and neighbors as their primary source of information about conservation, but unless the friend or neighbor is knowledgeable about conservation information, this information network is not as valuable at dispersing new information. The NRCS could consider ways to rely on the renters with whom they frequently interact to disseminate conservation information to their full-owner friends and neighbors. Ways could be found to financially and structurally support these farmer-to-farmer networks.

While some information about conservation practices seems to be diffusing effectively from NRCS staff to renters, there may be opportunities for heightening adoption of particular conservation practices by listening to the concerns of individual farmers about their particular farms and watersheds. It seems likely that the success of NRCS with renters comes from their familiarity with watershed renters, and the ongoing nature of this relationship. More likely than not, NRCS agents are listening to renters and designing on-farm solutions in light of particular conservation challenges. Likewise, owners are likely relying on neighbors and friends for conservation information because of the deep relationships between and among them. Besides neighbors and friends, we found that both owners and renters often rely on agencies like NRCS, the Farm Service Agency, and County Extension for conservation information. In light of that finding, local, state, and federal conservation agencies would be wise to build from what they already do well and from what farmers already need by creating context-specific mechanisms for supporting the combination of technical and practical expertise.

We emphasize the importance of building expert-farmer and farmer-to-farmer relationships to support conservation, in addition to more straightforward but passive mechanisms like brochures and mailings for delivering content about conservation practices from technical experts to farmers. This point is supported by our finding that both owners and renters rely on content delivery via magazines and printed materials, but also rely on interactive forums like meetings and field demonstrations and visits with trusted technical and local experts like NRCS, family members and neighbors, County Extension, and the Farm Service Agency. In order to deliver conservation information effectively to both owners and renters, the NRCS, Farm Service Agency, the County Extension Office, and other trusted, widely used information networks should utilize the sorts of personalized, interactive modes that farmers already depend on and trust.

We suggest that outreach agents focus on engagement, and work to adopt a contextual (Gross 1994) or deep communication (Druschke 2014) approach that attends to the characteristics of renters and full-owners, and considers how to engage farmers via communication efforts that speak in their terms and work in their particular landscapes. We hope outreach staff will continue to find novel ways to speak with farmers about conservation, and not just at them.

Contextual communication can be used to engage farmers who are concerned with both their pocketbooks and land conservation, as financial success is at the core of farm continuity. Inherently, farmers want to stabilize revenue through risk reduction, while concurrently maximizing profits. One way many farmers reduce risk is through excessive fertilizer application. By applying more fertilizer than is necessary, farmers reduce the risk of poor yield, therefore stabilizing revenue and potentially maximizing profits (Babcock and Shogren 1995). In this circumstance, contextual communication can provide dialogue with farmers about how their excess fertilizer ends up in waterways, thus reducing overall profits. When information about conservation is put into terms that engage farmers and their inherent risk reducing, profit-maximizing tendencies, we are apt to see more beneficial changes. Future quantitative and qualitative research should investigate farmers’ particular communication needs and interests, including explorations of the different terms, arguments, and beliefs that farmers in particular watersheds bring to bear on their conservation practice decisions, as both owners and renters (Druschke 2013).

In addition, farmers who pair technical knowledge from recognized experts with personal, hands-on expertise from friends and neighbors are able to build a knowledge foundation that they can apply to their own on-farm decisions (Rogers 2003). A difference in the use of information sources between renters and full-owners may be a key determinant in how and when each group adopts conservation practices. By revealing the types of conservation information sources used most frequently by each group, and identifying the adopters and non-adopters in each group, federal funding can be thoughtfully targeted towards non-adopters to increase adoption rates. Our study does not consider what drives a farmer who already owns some land to rent more farmland. There could be several reasons that correlate with information sources. For example, as one of the reviewers suggested, farmers who rent and own land could be more professionalized and therefore use different information channels. Future research should consider the motives behind farmers’ decisions to rent, how they relate to large scale trends in the agricultural sector such as land prices and farmers’ ages, and how they impact land management decisions.

We also note that adoption by both groups can be spurred by weather events. Druschke (2013) conducted in-depth interviews with farmers and conservation experts in this watershed and found that severe flooding in 2008 acted as a catalyst for the adoption of conservation practices. As weather events continue to increase in frequency and severity, adoption rates may increase because farmers will begin to see weather events as a pressing threat for accelerated erosion on their lands and realize the need for change. Understanding the role of adaptation to climate change in conservation practice adoption is an important future research need.

Our study found that even though trends are shifting and renters farm more acres while owners farm fewer, renters still adopt conservation practices at the same rate as owners. Renters also use conservation tillage significantly more often than owners, even though they plant corn more intensively. In addition, renters utilize more diverse information sources and communication channels than owners. Renters who adopt conservation practices are highly networked and not reliant on one single type of information, which indicates that they are more likely to be early adopters of other innovations. This adaptability and openness implies a promising future for the adoption of conservation practices and climate change adaptations, if local, state, and federal outreach staff can build from one-to-one, interactive networks and work with farmers to co-design socially and ecologically appropriate on-farm solutions.

While there is some evidence that renters participate in specific conservation programs more than owners (Kraft et al. 1996), participation in these programs is not consistent across programs (Reimer and Prokopy 2014). Meanwhile, the most recent Farm Bill enacted by the US Federal government has reduced, and in some cases eliminated, funding for many conservation programs (USDA Economic Research Service 2014). It is therefore essential to know how and why renters in the United States adopt conservation practices, how federal funding impacts the adoption of conservation practices, and who farmers look to for conservation knowledge in order to target future policies. Additionally, a limitation of our study is that it focuses on one watershed in Iowa. Future research could expand the geographical scope to include more states and watersheds. This would shed more light on how renters and owners across geographies view conservation practices and how they differ in their use of information sources and channels for conservation data.

Our results illustrate that there is not a simple connection between renters and conservation. We add to recent work that notes an increase in conservation practice adoption among tenants and part-owners, and consider the importance of these findings for communicating with owners and renters about conservation practices in the context of their particular concerns and needs. These results can be used by landowners and tenants to find common talking points and to guide lease agreements, and can be used by agencies working with full-owners, part-owners, and tenants to increase the adoption of conservation practices and, subsequently, the health of our agro-ecosystems.
